# Study on the relationship between obesity and complications of Pediatric Epilepsy surgery

**DOI:** 10.1186/s12887-023-03948-9

**Published:** 2023-03-30

**Authors:** Lei Shen, Mengyang Wang, Jingwei Zhao, Yuanyuan Ruan, Jingyi Yang, Songshan Chai, Xuan Dai, Bangkun Yang, Yuankun Cai, Yixuan Zhou, Zhimin Mei, Zhixin Zheng, Dongyuan Xu, Hantao Guo, Yu Lei, Runqi Cheng, Chuqiao Yue, Tiansheng Wang, Yunchang Zhao, Xinyu Liu, Yibo Chai, Jingcao Chen, Hao Du, Nanxiang Xiong

**Affiliations:** 1 Department of Neurosurgery, Zhongnan Hospital, Wuhan University, 430071 Wuhan, Hubei China; 2 Department of Neurosurgery, Wuhan Children’s Hospital, 430010 Wuhan, Hubei China

**Keywords:** BMI, Intraoperative blood loss, Obesity, Pediatric epilepsy, Postoperative fever, Refractory epilepsy

## Abstract

**Objective:**

Studies have shown that obesity has a significant impact on poor surgical outcomes. However, the relationship between obesity and pediatric epilepsy surgery has not been reported. This study aimed to explore the relationship between obesity and complications of pediatric epilepsy surgery and the effect of obesity on the outcome of pediatric epilepsy surgery, and to provide a reference for weight management of children with epilepsy.

**Methods:**

A single-center retrospective analysis of complications in children undergoing epilepsy surgery was conducted. Body mass index (BMI) percentiles were adjusted by age and used as a criterion for assessing obesity in children. According to the adjusted BMI value, the children were divided into the obese group (n = 16) and nonobese group (n = 20). The intraoperative blood loss, operation time, and postoperative fever were compared between the two groups.

**Results:**

A total of 36 children were included in the study, including 20 girls and 16 boys. The mean age of the children was 8.0 years old, ranging from 0.8 to 16.9 years old. The mean BMI was 18.1 ^kg/m2^, ranging from 12.4 ^kg/m2^ to 28.3 ^kg/m2^. Sixteen of them were overweight or obese (44.4%). Obesity was associated with higher intraoperative blood loss in children with epilepsy (p = 0.04), and there was no correlation between obesity and operation time (p = 0.21). Obese children had a greater risk of postoperative fever (56.3%) than nonobese children (55.0%), but this was statistically nonsignificant (p = 0.61). The long-term follow-up outcomes showed that 23 patients (63.9%) were seizure-free (Engel grade I), 6 patients (16.7%) had Engel grade II, and 7 patients (19.4%) had Engel grade III. There was no difference in long-term seizure control outcomes between obese and nonobese groups (p = 0.682). There were no permanent neurological complications after surgery.

**Conclusion:**

Compared with nonobese children with epilepsy, obese children with epilepsy had a higher intraoperative blood loss. It is necessary to conduct early weight management of children with epilepsy as long as possible.

## Background

Epilepsy is one of the most common disabling chronic neurological diseases [1]. Despite the availability of over 20 antiepileptic drugs (AEDs) for the symptomatic treatment of epilepsy, approximately one-third of patients with epilepsy have epilepsy refractory to AEDs [2]. It is generally acknowledged that epilepsy surgery and neuromodulation surgery are effective therapies to treat refractory epilepsy [3, 4]. Recent studies have shown that neurostimulation has also become one of the effective therapies for refractory epilepsy [5, 6].

Childhood obesity is one of the primary public health problems faced by children [7]. Recent surveys show that 17.1% of children have obesity, with an increasing obesity rate of children [8, 9]. Obesity is particularly common in children with epilepsy due to the side effects of AEDs [10, 11]. It has been reported that 38.6% of children with epilepsy are overweight or obese, of which 19.9% are obese and 18.7% are overweight [12].

Studies have shown that obesity is one of the important risk factors for poor surgical outcomes, which may be related to prolonged operation time, poor wound healing, and comorbidities in obese patients [13–16]. However, the relationship between obesity and pediatric epilepsy surgery has not been reported. By reviewing the cases of children with refractory epilepsy, this study discussed the relationship between obesity and complications of pediatric epilepsy surgery with refractory epilepsy and the effect of obesity on the outcome of pediatric epilepsy surgery, and provided a reference for weight management of children with epilepsy.

## Methods

### Case selection

Data from patients with refractory focal epilepsy who underwent epilepsy surgery in Wuhan Children’s Hospital from January 2017 to October 2021 were collected. Epilepsy surgery included temporal lobectomy, selective amygdalohippocampectomy, selective amygdalohippocampotomy, frontal lobotomy, and hemispherotomy. Inclusion criteria included the following: (1) Patients were diagnosed with refractory focal epilepsy. (2) The patients were younger than 18 years old at surgery. Exclusion criteria included the following: (1) The necessary clinical data of cases were incomplete, including the height and weight of patients. (2) The presence of intraoperative blood loss and postoperative fever could not be determined or recorded.

### Extraction of clinical data

The clinical data, including demographic characteristics, operation time, intraoperative blood loss, and postoperative fever, were extracted from electronic health records. The amount of intraoperative blood loss was measured by the suction device: the container of the suction device had a scale to measure the amount of imbibition. Meanwhile, the amount of saline used for intraoperative irrigation was also recorded. Then the difference between the amount of imbibition in the container of the suction device and the amount of saline was considered as the amount of intraoperative blood loss. The measurements of height and weight were as follows. For children up to 2 years old, a length measuring device was used to measure length as height and a horizontal baby electronic scale was used to measure weight. For children over 2 years old, a standing height meter was used to measure height and a vertical weight scale was used to measure weight.

The formula for calculating body mass index (BMI) was BMI = weight/height^2^. BMI percentiles adjusted for age were used as a criterion for assessing obesity in children [17]. As a secondary response, BMI percentiles were classified into the following categories: (1) obese: The BMI was ≥ the 85th percentile for age, (2) overweight: The BMI was more than 85th percentile and less than the 95th percentile for age, (3) nonobese: The BMI was<the 85th percentile for age. In order to facilitate the comparison and analysis, cases were divided into an obese group (obese or overweight cases) and a nonobese group (nonobese cases) by BMI percentiles.

### Follow-up

Follow-up methods mainly included outpatient follow-up and telephone follow-up. All patients were followed up for at least 12 months. Postoperative epilepsy control was assessed by Engel classification.

### Statistical analysis

Descriptive statistics were used to describe the group characteristics of children. Continuous variables included the following: The D’Agostino-Pearson normality test was used to assess whether the intraoperative blood loss followed a normal distribution, and the unpaired t-test was used to compare whether there was a difference in intraoperative blood loss between obese and nonobese groups. The mean ± standard deviation and 95% confidence intervals (CIs) were used to measure the size of the difference between groups. Discrete variables included the following: Fisher’s exact test was used to compare whether there was a difference in the incidence of postoperative fever between obese and nonobese groups. All tests were 2-sided and used a 0.05 significance level.

## Results

### Clinical data

A total of 36 cases were included in the study. The clinical data of the children are shown in Table 1, including 20 male children and 16 female children. The mean age of the children was 8.0 years old, ranging from 0.8 to 16.9 years old. The mean BMI was 18.1 ^kg/m2^, ranging from 12.4 ^kg/m2^ to 28.3 ^kg/m2^. Five patients were overweight (13.9%), and 11 patients were obese (30.6%). The epilepsy surgeries included temporal lobectomy (n = 5), selective amygdalohippocampectomy (n = 6), selective amygdalohippocampotomy (n = 8), frontal lobotomy (n = 2) and hemispherotomy (n = 15), with no significant difference in the distribution between groups (p = 0.91). There were no wound complications in two groups.

**Table 1:** Clinical data of the collected cases. SAHC: Selective Amygdalohippocampectomy; SAHCo: Selective Amygdalohippocampotomy

	Total(n = 36)	Nonobese (n = 20)	Obese(n = 16)	P value
**Age(y)**	8.0 ± 4.5	8.3 ± 4.4	7.6 ± 4.6	0.67
**Male/Female**	20/16	13/7	7/9	0.31
**BMI**	18.1 ± 3.8	15.8 ± 1.9	20.9 ± 3.6	< 0.01
**Surgical technique**				0.91
Temporal lobectomy	5	2	3	
SAHC	6	4	2	
SAHCo	8	5	3	
Frontal lobotomy	2	1	1	
Hemispherotomy	15	8	7	
**Operation time (min)**	188.2 ± 59.5	200.6 ± 47.4	171.7 ± 69.2	0.22
**Intraoperative blood loss(ml)**	136.2 ± 85.4	104.3 ± 58.6	173.3 ± 96.1	0.04
**Postoperative fever**				0.61
No fever	16	9	7	
0–2 days	8	3	5	
3–6 days	4	3	1	
7–10 days	8	5	3	
**Intracranial infection**	0	0	0	> 0.99

### Obesity and intraoperative blood loss

**Fig. 1:**
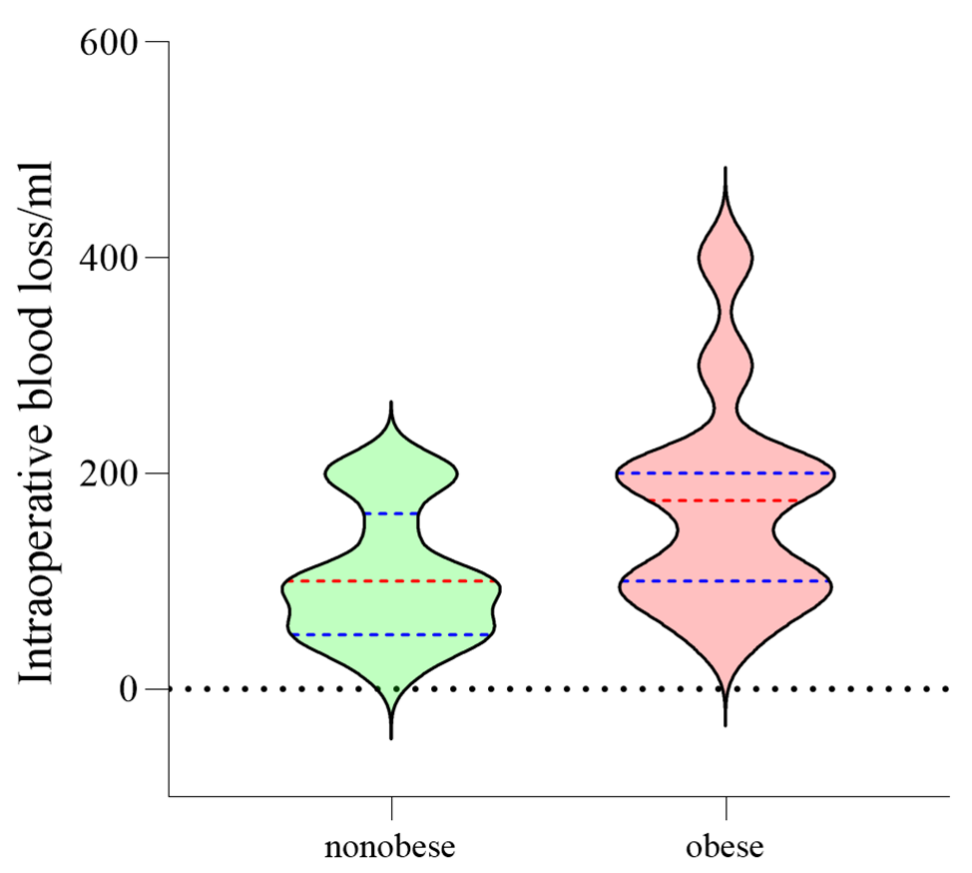
A violin diagram showed that the overall distribution of the obese and nonobese groups was similar, and the intraoperative blood loss in the obese group was higher than that in the nonobese group

The mean intraoperative blood loss in the obese group was 173.3 ± 96.1 ml, ranging from 56.0 to 397.0 ml. The mean intraoperative blood loss in the nonobese group was 104.3 ± 58.6 ml, ranging from 22.0 to 208.0 ml. The D’Agostino-Pearson normality test showed that the data in both groups followed a normal distribution (obese group: p = 0.15; nonobese group: p = 0.44). The mean difference between the two groups was 69.1 ± 32.0 ml, 95% CI: 3.0-135.1 ml. The results of the unpaired t-test showed that the difference was statistically significant (p = 0.04) (Fig. 1).

### Obesity and operation time of children

**Fig. 2:**
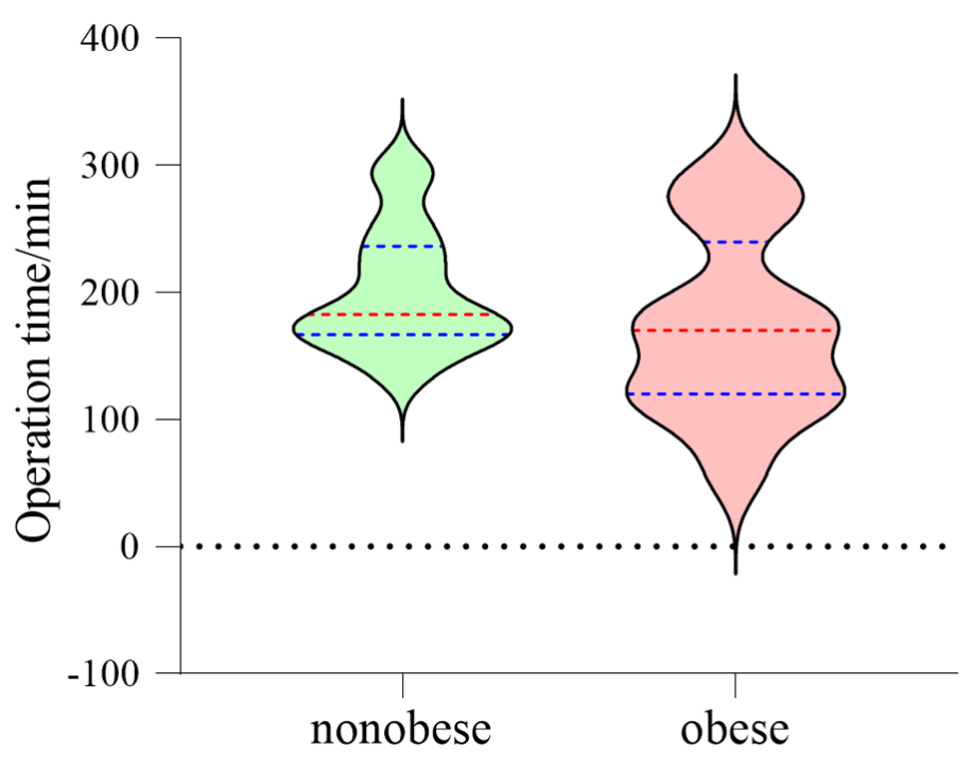
A violin diagram showed that the overall distribution of obese and nonobese groups was similar, and there was no significant difference between the two groups

The mean operation time of the obese group was 171.7 ± 69.2 min, ranging from 63.0 to 294.0 min. The mean operation time in the nonobese group was 200.6 ± 47.4 min, ranging from 155.0 to 303.0 min. The D’Agostino-Pearson normality test showed that the two groups of data followed a normal distribution (obese group: p = 0.69; nonobese group: p = 0.31). The mean difference between the two groups was 29.0 ± 22.9 min, 95% CI: 18.1–76.0 min. Unpaired t-test results showed that the difference was not statistically significant (p = 0.22) (Fig. 2).

### Obesity and postoperative fever in children

According to the occurrence time of postoperative fever, postoperative fever was divided into four groups: no fever, early postoperative fever (0–2 days), middle postoperative fever (3–6 days), and late postoperative fever (7–10 days).

**Fig. 3:**
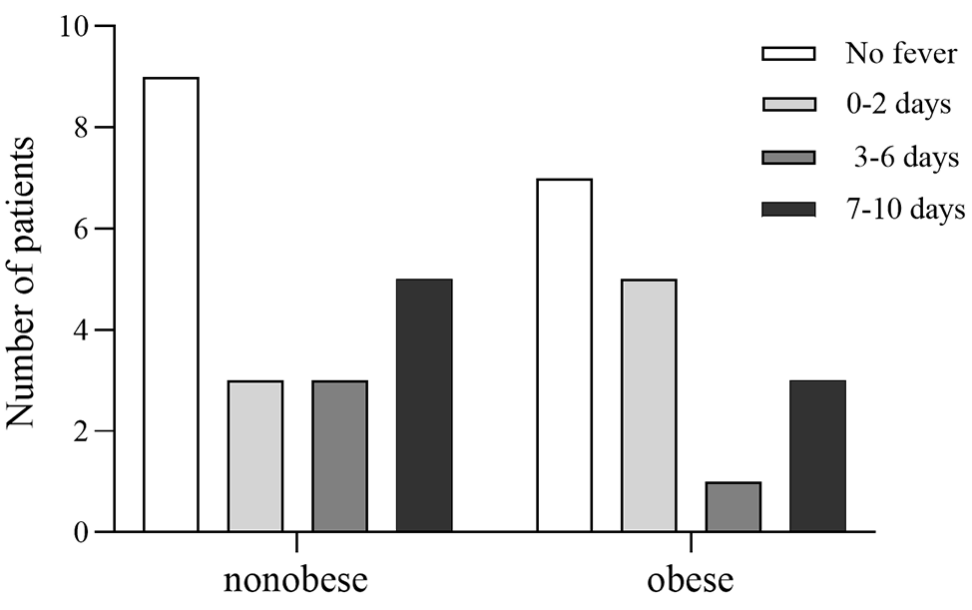
Interleaved bars showed that the obese group had a higher incidence of postoperative fever than the nonobese group

The incidence of postoperative fever of patients was shown in Table 1. A total of 20 patients (55.6%) had a postoperative fever, 9 (56.3%) in the obese group and 11 (55.0%) in the nonobese group. Among them, 8 patients had an early postoperative fever, 5 in the obese group and 3 in the nonobese group; 4 patients had a middle postoperative fever, 1 in the obese group and 3 in the nonobese group; and 8 patients had a late postoperative fever, 3 in the obese group and 5 in the nonobese group. The obese group had a higher incidence of postoperative fever than the nonobese group, but Fisher’s exact test showed that obesity was not significantly associated with postoperative fever (p = 0.61) (Fig. 3).

### Follow-up

The mean follow-up time of the patients was 20 months, ranging from 12 to 48 months. The long-term follow-up outcomes showed that 23 patients (63.9%) were seizure-free (Engel grade I), 6 patients (16.7%) had Engel grade II, and 7 patients (19.4%) had Engel grade III. In the obese group, 10 patients had Engel grade I, 2 had Engel grade II, and 4 had Engel grade III. Meanwhile, 13 patients had Engel grade I, 4 had Engel grade II, and 3 had Engel grade III in the nonobese group. There was no difference in long-term seizure control outcomes between obese and nonobese groups (p = 0.682). In addition, there were no long-term neurological complications after surgery, such as aphasia and hemiplegia.

## Discussion

### Obesity and epilepsy

It has been reported that 38.6% of children with epilepsy are overweight or obese, of which 19.9% ​​are obese and 18.7% are overweight, more than double the proportion of children expected to be overweight in a normal population [12]. Due to the side effects of AEDs such as valproic acid, carbamazepine, and gabapentin, taking AEDs may lead to obesity in children. At the same time, obesity in children can also lead to a decrease in their medication compliance, which further leads to poor epilepsy control [18–20]. Among the cases included in our study, 13.9% were overweight and 30.6% were obese, which is consistent with Daniels’ results [12]. Regardless, it is necessary to conduct early control and treatment of epilepsy in children in time and to control the weight of children with epilepsy as long as possible.

### Obesity and intraoperative blood loss and operation time

Studies have shown that obesity is a risk factor for poor surgical outcomes [13–16]. Obesity is an independent risk factor for prolonged operation time and room time [21], postoperative thrombotic complications [22], atrial arrhythmias [23, 24], and wound infection [25].

We found that the mean intraoperative blood loss in the obese group was significantly higher than that in the nonobese group (p = 0.04). Tjeertes found higher intraoperative blood loss in obese patients, possibly because obese patients had more difficulty in exposing and dissecting the surgical site, requiring more tissue to be cut, prolonging operation time, and increasing intraoperative blood loss [26]. The operation time may change according to surgical technique and practitioner. In our study, all epilepsy surgeries were performed by the same neurosurgeon, and there was no difference in the distribution of different epilepsy surgeries among the cases according to Table 1(p = 0.91), so the possible influence of surgical technique and practitioner on the operation time could be excluded to some extent. However, our study found no significant difference in operation time between obese and nonobese groups (p = 0.22), suggesting that the length of operation time was not responsible for the difference in intraoperative blood loss between the obese and nonobese groups. Similarly, the possible influence of age and surgical technique on intraoperative blood loss could be partially excluded.

Furthermore, coagulopathies, especially thrombocytopenia, are considered as the side effects of some AEDs so that patients on these AEDs might have a bleeding tendency. Gerstner found that valproate-associated coagulopathies were frequent and variable in children [27]. Another study showed that valproic acid was associated with a decreased platelet count, although thrombocytopoiesis is not affected, even in children with a reduced platelet count [28]. Carbamazepine was also thought to cause thrombocytopenia besides valproic acid through an autoimmune mechanism [29, 30]. Based on the NICE guideline [NG217] (https://www.nice.org.uk/guidance/ng217), lamotrigine and levetiracetam were considered as the first-line AEDs for focal epilepsy rather than valproic acid. For patients with refractory focal epilepsy in our study, we usually used a first-line AED combined with one of oxcarbazepine, perampanel, and nitrazepam according to the patient’s specific condition. If the patient was on valproic acid before being hospitalized, it would be stopped and be switched to oxcarbazepine for at least one week before the surgery.

In addition, there may be another reason that adipose tissue in obese children mainly accumulates in the trunk and limbs, while the epilepsy surgery site is in the brain, and there is little fat accumulation at the surgery site. Therefore, for children with epilepsy, higher intraoperative blood loss in the obese group may not be associated with operation time. Some studies have found that obese patients have a hyperactive inflammatory response, increased angiogenesis in the tissue compared with nonobese patients, a more abundant blood supply in the tissue, a higher bleeding risk, and a higher amount of intraoperative blood loss [31–33].

### Obesity and postoperative fever

Fever is one of the most common postoperative complications of surgery. According to the cause of fever, postoperative fever can be divided into infectious fever and non-infectious fever. Non-infectious fevers are in turn associated with trauma and inflammation from the surgery, suture foreign body reactions, transfusion reactions, and drug-induced fevers [34, 35]. According to the time postoperative fever occurs, postoperative fever can be divided into early postoperative fever (0–2 days), middle postoperative fever (3–6 days) and late postoperative fever (7–10 days).

A total of 20 patients (55.6%) had a postoperative fever, 9 patients (56.3%) in the obese group and 11 (55.0%) in the nonobese group. The incidence in the obese group was slightly higher than that in the nonobese group, and the obese group tended to have early postoperative fever, while the nonobese group was more likely to have middle-late postoperative fever; however, the statistical results showed no significant correlation between obesity and postoperative fever (p = 0.61). There was no difference in postoperative fever between obese and nonobese groups in our study. The possible reason is that the type of postoperative fever in the children was mainly non-infectious fever caused by surgical trauma.

It has been reported that obese patients have lower immunity and are more likely to have postoperative infectious fever [36–38]. Meanwhile, obesity has been found to be associated with altered collagen structure and resistance to leptin, leading to impaired wound healing [39]. However, all cases included in our study had good postoperative wound healing and no intracranial infection, which means that postoperative fever was not associated with surgical site infection.

Intraventricular blood loss after neurosurgery is a recognized reason for aseptic meningitis and non-infectious fever [40]. There was a significant difference in intraoperative blood loss between obese and nonobese groups in our study, while no significant difference was found in the incidence of postoperative fever between the two groups. Almeida found that ventriculotomy is not an independent cause of fever after hemispherectomy [41], probably because intraventricular blood loss after ventriculotomy can be well managed and an insufficient amount of blood remains to cause a postoperative fever.

### Limitations

This study still has some limitations. First, this study was a retrospective study and it was subject to inherent bias in the study design. Also, epilepsy surgery has different surgical approaches depending on the etiology and localization of epileptogenic foci, and it is difficult to determine whether different surgical approaches will affect intraoperative blood loss and postoperative fever. In addition, the sample size of cases and observation indicators included in this study were small. Considering that the immune system of children has not yet been established, they are more likely to have a fever with unknown causes. Prospective studies with larger sample sizes are still required to further explore the relationship between obesity and epilepsy surgical complications in children.

## Conclusion

This study investigated the relationship between obesity and intraoperative blood loss and postoperative fever in children with refractory epilepsy. Compared with nonobese cases, obese cases had higher intraoperative blood loss during surgery. Obesity was not associated with postoperative fever or operation time. It is necessary to conduct early weight management of children with epilepsy as long as possible. Due to the small sample size of cases and limited observation indicators included in this study, the relationship between obesity and complications of pediatric epilepsy surgery still needs to be further explored.

## Data Availability

The clinical data of patients in the current study were extracted from electronic health records in the medical record system of Wuhan Children’s Hospital. The clinical data could be obtained through Dr. Ruan at Wuhan Children’s Hospital.
